# Survey of patient and public perceptions of electronic health records for healthcare, policy and research: Study protocol

**DOI:** 10.1186/1472-6947-12-40

**Published:** 2012-05-23

**Authors:** Serena Luchenski, Anjali Balasanthiran, Cicely Marston, Kaori Sasaki, Azeem Majeed, Derek Bell, Julie E Reed

**Affiliations:** 1 Imperial College London, South Kensington Campus, London, SW7 2AZ, UK; 2 London School of Hygiene and Tropical Medicine, 15-17 Tavistock Place, London, WC1H 9SH, UK

**Keywords:** Electronic Health Records, Patient and Public Perceptions, Quality Improvement

## Abstract

**Background:**

Immediate access to patients’ complete health records via electronic databases could improve healthcare and facilitate health research. However, the possible benefits of a national electronic health records (EHR) system must be balanced against public concerns about data security and personal privacy. Successful development of EHR requires better understanding of the views of the public and those most affected by EHR: users of the National Health Service. This study aims to explore the correlation between personal healthcare experience (including number of healthcare contacts and number and type of longer term conditions) and views relating to development of EHR for healthcare, health services planning and policy and health research.

**Methods/design:**

A multi-site cross-sectional self-complete questionnaire designed and piloted for use in waiting rooms was administered to patients from randomly selected outpatients’ clinics at a university teaching hospital (431 beds) and general practice surgeries from the four primary care trusts within the catchment area of the hospital. All patients entering the selected outpatients clinics and general practice surgeries were invited to take part in the survey during August-September 2011. Statistical analyses will be conducted using descriptive techniques to present respondents’ overall views about electronic health records and logistic regression to explore associations between these views and participants’ personal circumstances, experiences, sociodemographics and more specific views about electronic health records.

**Discussion:**

The study design and implementation were successful, resulting in unusually high response rates and overall recruitment (85.5%, 5336 responses). Rates for face-to-face recruitment in previous work are variable, but typically lower (mean 76.7%, SD 20). We discuss details of how we collected the data to provide insight into how we obtained this unusually high response rate.

## Background

Universal electronic health records (EHRs) appear to be an inevitable technological advance in the computerised communications age. Nationally linked EHR systems remain relatively rare, but are being developed around the world, including Canada [1], Australia [2], the United States [3], and the United Kingdom [4]. Among the potential benefits of storing and sharing patient information electronically are: improved legibility and better access to medical records, improvement in the quality and safety of medical services, reduction in healthcare costs, improved population-level health and enhanced health research [3,5,6]. However, there are also potential risks which must be mitigated if EHRs are to be publically accepted and universally implemented [3].

Some of the ethical and political challenges presented by EHRs include citizens’ rights over ownership of their own medical data, specifically on what terms and in what ways personal or anonymised health records can be used for medical treatment and research. Furthermore, there are concerns about the security of electronic databases and the socio-political implications of such a database for the ‘surveillance society’ ([7-9]; see also [10]). The issues around personal privacy and security are complex and may present barriers to public acceptance of storing and sharing personal health information [3].

In the UK a national policy, *Connecting for Health*, was developed with the primary purpose of developing a computer database of patient health records ‘from cradle to grave’ for use by clinicians [11]. Connecting for Health also included a Research Capability Programme that aimed to ensure that researchers had access to patient data, subject to ethical and legal protections. The current UK coalition government now plans to keep the existing infrastructure and try to support and connect local records systems, rather than continuing to develop a standardised national EHR system (for a brief history see [4]). The future of EHRs in the UK is a subject of much political debate, but the perspective of the principal consumers of health records for medical care – patients and members of the public – is often missing from the discussion.

Few studies have examined patient and public perceptions about electronic health data and universal EHR systems, and these have focused on individuals’ fears and concerns about EHRs, rather than any hopes they might have for improvement in their own health care and public health more broadly. Previous studies in the general population have found that UK adults [12] and young people [13] have concerns about using EHRs for medical research. Their concerns were about privacy, security, control over access, and utilization and misuse of data. However, when asked about cancer specifically [14], people were in favour of using electronic records for public health research and surveillance. Those with long-term conditions and higher levels of literacy about health and healthcare also tend to be more in favour of the use of electronic records (summary care records) than those without such conditions or little knowledge or experience of healthcare issues [15]. Views about EHRs are therefore likely to differ according to personal circumstances, such as medical status, age, socioeconomic position, and previous healthcare experiences, and according to the proposed usages of EHRs. This study seeks to extend these findings by examining additional patient and public characteristics, including health services use, and by examining a broad range of attitudes towards a universal EHR system.

### Objective

The aim of this study is to enhance current understanding of patient and public views about the development of universal patient EHRs for healthcare and research. First, we will investigate the level of acceptance for a national EHR system and for using EHRs for healthcare, health services planning and policy and health research. Second, we will examine how these broad views are correlated with: individuals’ personal experiences of healthcare and research; the number and type of long-term conditions respondents have; and individuals’ sociodemographic characteristics. Finally, we will examine individuals’ specific hopes and concerns about EHRs to gain a deeper understanding of patient and public views about EHRs.

## Methods/design

### Study and questionnaire design

We conducted a cross-sectional self-complete questionnaire survey using a stratified cluster random sample of 5336 patients and members of the public in an area of northwest London, UK. Before administration, the questionnaire was designed using *Cardiff TELEform* survey software and piloted for use with 30 adults (over age 18) from the general population. Pilot participants were selected from patient advocacy groups, patient and public involvement networks, and personal contacts of the research team. Participants varied according to age, gender, education, ethnicity, parents, carers, people with and without long-term health conditions, and people with differing levels of experience with healthcare practice and research. Multiple rounds of piloting and revision of the questionnaire were conducted over a period of three months until all participants fully understood each question, accepted the design and layout as a whole, and were able to complete it within ten minutes. This study was granted ethical approval from the National Research Ethics Service in Dulwich, London.

### Setting and sampling

The cross sectional survey was conducted over six weeks from 1 August – 9 September, 2011. We invited potential participants from waiting rooms of eight outpatient clinics at a university teaching hospital with 431 beds in northwest London, and eight general practice (GP) surgeries from the four boroughs within the catchment area of the hospital (Kensington and Chelsea, Wandsworth, Hammersmith and Fulham, or Westminster). This design was chosen to maximise variability in patients’ healthcare experiences in order to investigate potential associations with their views about electronic health records. We hypothesised outpatients as a population to have more complex health needs and potentially to have had more contact with the healthcare system, whereas GP patients as a population were expected to be healthier with fewer healthcare contacts.

Each of the eight hospital outpatients’ clinics were sampled on five days (one Monday, one Tuesday, one Wednesday, one Thursday and one Friday), totalling 40 outpatients’ clinic sampling days. The specific date when each clinic was visited was randomly selected over the 6 week recruitment period. This design was chosen to ensure a wide array of patient characteristics and to minimise selection bias. For GP surgeries, we used the complete list of GP surgeries in the catchment area, stratified by borough, size and whether or not they were research active, to select a random sample of surgeries. We selected one large surgery (patient list ≥ 5000) and one small surgery (patient list <5000) from each borough, for a total of eight surgeries. Only surgeries listed as willing to participate in research were chosen because of resource constraints for recruitment. Six GP surgeries were selected but refused to participate. We replaced these with additional surgeries using the same selection method. Recruitment was again conducted on five randomly selected week days for each surgery over the period, for a total of 40 GP surgery sampling days.

### Participants and data collection

Our recruitment team consisted of a lead project coordinator, two assistant coordinators and six student research assistants (RA), five of whom were medical students. Teams of two RAs were present throughout the entire working day of each clinic or surgery sampling day. Every person entering the waiting room was invited to take part in the survey and the number and gender of refusals was recorded.

Eligibility criteria for participation were: a) 18 years or older); b) first time filling in the survey; and c) able to understand the information describing the research study. The first page of the questionnaire detailed the nature of the study, asked participants to confirm their eligibility and to provide informed consent prior to filling in the survey. The RAs were available to answer any questions.

The RAs collected the completed questionnaires from participants and returned them to the project office where they were scanned. The data were automatically converted into an electronic dataset using *TELEform* software. Consent and potential data errors highlighted by the software were checked manually by the project coordinators. In effect, this method is equivalent to ‘double data entry’.

### Study size

In this exploratory study, formal sample size calculations were not appropriate as previous studies to inform the calculation were not available. According to Harrell’s rule of thumb [16], there should be at least ten individuals per candidate independent variable to conduct multiple logistic regression, and thus a minimum of 600 participants for our study. However, we decided to recruit a larger sample to allow subgroup analyses. For instance, if we decided to stratify the sample by age (50 or less, over 50) and 50% of the sample was over 50, we would then need 1200 participants to be sufficiently powered using the rule of thumb. Thus, given the variety of subgroup analyses possible with this sizeable dataset, we decided to take a pragmatic approach and recruit the largest sample we could within the constraints of the project.

Based on previous experience of the researchers working within the sector, we anticipated that it would be possible to recruit a conservative average of 50 patients per sampling day in each outpatient clinic and GP surgery using the method described above. Thus, we aimed to recruit 4000 participants in total (50 respondents × (40 outpatients days + 40 GP days)) which would be a large enough sample to conduct most subgroup analyses, depending on the number of respondents in each category. In practice, we recruited an average of 89 people per day for a total of 5336 respondents. The response rate was 85.5%.

#### Variables

The main variables collected were on patient and public hopes and concerns relating to their own participation in a national EHR system. These concepts were assessed using several questions in the questionnaire (see Additional file 1).

#### Patient and public views about EHRs

Participants were asked: if there were a national EHR system, would they want their record to be a part of the system for their own personal healthcare, for health services planning and policy, and for health research. For healthcare, participants were asked to choose whether records should be available on a complete or incomplete basis. With respect to health services planning and policy and health research, these questions were asked in relation to anonymous and personalised records. Finally, respondents were asked if overall they supported the development of a national EHR system. In addition, we asked questions regarding access to and security of EHRs as these views may underlie, at least in part, the broad views of a national EHR system as described above.

For access, respondents were asked about their views about access to their complete and partial EHR as well as their anonymous (name and address removed) and personalised (name and address present) record. The questions referred specifically to the following groups: doctors and nurses, pharmacists, GP receptionists, patients (accessing their own record), NHS managers, health policy makers, NHS researchers, academic health researchers, health charities and drug companies.

Regarding security, respondents were asked about their views about the level of potential security risks to a future national EHR system and whether or not they thought the UK National Health Service was capable of securing EHRs. They were then asked to compare the security risks of a national EHR system to the current records system and then to indicate whether they felt they would be worried about the security of their record if there was a national EHR system. Finally, they were asked if their record was part of an EHR system whether or not they would choose to be asked first before their records were accessed for any reason.

#### Previous healthcare and research experience, long-term conditions, and sociodemographic characteristics

Questions about previous experience of the healthcare system and health research, long-term conditions, and sociodemographic characteristics were asked with the aim of examining their associations with participants’ views, as described in the previous section. Questions about patient and public experience of the healthcare system related to: working in healthcare, previous participation in a research study, satisfaction with healthcare received, previous exposure to EHRs, and the locations and number of times where an individual has accessed healthcare personally, as a parent and as a carer. Long-term conditions were assessed using a single question which asked respondents to mark all of the conditions that they have. Whether or not respondents had any condition, the number of long-term conditions they had, and whether or not they had any conditions with negative social implications, e.g. alcohol, drug, or mental health problems, were derived and considered the main variables of interest, although analyses of specific conditions will be possible with the data. Birth year, gender, ethnicity, highest level of education, confidence using computers and borough of residence were also collected. Again, further details of measurement are provided in the Additional file 1.

### Statistical analysis plan

We will conduct three main analyses corresponding to our objectives, but given the size of the dataset there will be scope for subsequent secondary analysis. The first will be a descriptive analysis in which we will examine the proportions of respondents who would support the development of a national EHR system for the UK and the proportions of respondents who think EHRs should be used for their own personal healthcare, health services planning and policy and health research. We will also examine whether respondents preferred anonymous EHRs to be used or whether they thought EHRs should contain complete or partial information about the patient.

The aim of the second analysis will be to describe associations between the views found in the first analysis with individuals’ personal healthcare and research experiences, their long-term conditions, and their sociodemographic characteristics using bivariate multinomial regression. In addition, we will build a multivariate multinomial regression model of best fit to examine the variability in patient and public views about EHRs.

In the third main analysis we will use bivariate and multivariate multinomial regression to explore how individuals’ views about access, security and personal choices relating to EHRs are associated with their broad views examined in the first two analyses (overall support of a national EHR system, using EHRs for personal healthcare, health services planning and policy and health research).

To support each of these analyses, we will also conduct an analysis of missing data. We will describe the sample by calculating the proportions of responses and non-responses for each variable and for the questionnaire overall and then we will investigate whether patterns of missing data vary according to key sociodemographic characteristics. Only complete cases will be included in each analysis and respondents will be excluded using listwise deletion. Given the richness of this dataset, we expect there to be further questions worthy of investigation and will thus conduct further analyses as appropriate. We will use the ‘vce’ procedure in Stata to account for the clustered sampling design for each analysis. Alpha-level 0.05 will be used to determine statistical significance.

## Discussion

Response rates and overall recruitment for this study exceeded expectations. On average, 89 people were recruited each day for a total of 5336 respondents (average response rate 85.5%). A review of published patient satisfaction studies found that typical response rates for a face-to-face approach for participant recruitment are around 76.7% (SD 20, n = 61) [17]. We discuss some of the practical aspects of this study which may have contributed to the higher than usual response rate observed.

Before recruitment began we developed a comprehensive communication strategy. Our large multi-site survey needed the acceptance and cooperation of the many individuals working in participating outpatient clinics and GP surgeries. We identified those that would be most affected by the presence of our study, including frontline staff and managers, and used our coordination team members to raise awareness about the study, via individual discussions, departmental presentations and notices posted in the trust bulletins and on email lists.

A good team of research assistants is essential for successful recruitment and high quality data collection. We recruited and trained all the RAs for five days, arranged meetings with frontline staff before recruitment began, and met the RAs every day during fieldwork to ensure they received adequate support. To help with motivation, we implemented a popular system where the team with the highest number of recruited participants were offered the opportunity to deliver a scientific presentation about the study at a local research meeting in the hospital.

Thus, the way the study was organised may have contributed to the high response rates observed. Another possible contributor was the fact that stories about EHRs appeared in the UK media several times during recruitment, which may have increased interest in the topic. The physical characteristics of the outpatient and GP environments and waiting times may also have affected our response rates. Finally, characteristics of the research team or the participants themselves may have made patients particularly willing to respond. For instance, previous research has found that patients are keen to participate in research studies when approached by a reputable academic establishment [18].

## Conclusions

This large scale cross-sectional survey will examine perceptions of and attitudes towards uses of electronic patient health information in London. Associations with patient and public views and their personal circumstances and experiences have not been previously explored. Findings from this study will help inform UK policy on electronic health records.

## Abbreviations

EHR, Electronic health record; GP, General practice.

## Competing interests

The authors declare that they have no competing interests.

## Authors’ contributions

SL designed the questionnaire, sampling method and analysis plan and drafted the manuscript. AB coordinated the data collection and helped to draft the manuscript. CM contributed to the design of the study and data collection tools and reviewed the manuscript. AM contributed to the design and coordination of the study and reviewed the manuscript. KS contributed to the design and coordination of the study and reviewed the manuscript. DB conceived of the study and contributed to its design and coordination and reviewed the manuscript. JR conceived of the study and provided oversight to its design and coordination and contributed to the manuscript. All authors read and approved the final manuscript*.*

## Pre-publication history

The pre-publication history for this paper can be accessed here:

http://www.biomedcentral.com/1472-6947/12/40/prepub

## Supplementary Material

Additional file 1 Patient questionnaire. Attach PDF questionnaire as an appendix. Click here for file
