# Supplementary material for: Survey of patient and public perceptions of electronic health records for healthcare, policy and research: Study protocol
Source: BMC Med Inform Decis Mak. 2012 May 23;12:40. doi: 10.1186/1472-6947-12-40 (PMC3464182; doi:10.1186/1472-6947-12-40)
Supplement: Additional file 1 — Patient questionnaire. Attach PDF questionnaire as an appendix. [file 1472-6947-12-40-S1.pdf]

PATIENT SURVEY

Can you please spare 10 minutes to let us know your views about how the NHS should manage your health records in our computer age?

Please only take part in this survey once and only if you are over the age of 18.

We are independent university researchers from Imperial College, London. We are working with Chelsea and Westminster Hospital to research public and patient views about the use of computers to store patient records for healthcare and research. We are asking members of the public in Northwest London to tell us about their ideas. We will use the information to help inform what happens in the future.

This survey is about 'Electronic Health Records'. If created, your electronic health record would store everything about your health and the healthcare you receive from your birth until your death. Electronic health records would bring together in one record all of your separate files, whether stored on paper or a computer, in all of the different locations where you get healthcare. There are many arguments for and against electronic health records and we would like to know your views about this.

The NHS has started making electronic 'Summary Care Records' which you may have heard about. These are summaries about your health (e.g. medications and allergies), rather than complete health records like electronic health records. ***We are not asking about Summary Care Records***.

This questionnaire will take about 10 minutes.

All information will be kept confidential and your name will not be recorded. We will not be able to link your responses back to you.

If you would like to learn more about the study, please ask a member of our research team.

Thank you for participating in this study.

Professor Derek Bell and Ms Serena Luchenski

PLEASE COMPLETE THIS SECTION BEFORE YOU FILL IN THE QUESTIONNAIRE:

I confirm that I am ***over 18*** years of age and that this is the ***first time*** I am filling in this survey. My participation is voluntary and I may choose not to answer some questions or to stop answering the questionnaire at any point without giving a reason. I confirm that I have read and understood the above information.

Please place an X in the most appropriate box.

☐ **Yes, I will participate**

☐ **No, I do not wish to participate**

First of all, we'd like to know about your experience.

1. Have you ever worked in a job related to healthcare?

- ☐ No PLEASE SKIP TO QUESTION 2
- ☐ Yes

If yes, in which jobs? *Please X all that apply*

- ☐ Provider (e.g. nurse, doctor, etc.)
- ☐ Researcher (e.g. NHS, university, public health, etc.)
- ☐ Another job (e.g. GP receptionist, hospital manager, health charity, pharmacist, etc.)

2. Overall, how satisfied are you with the care you have received in the NHS?

☐ Very satisfied ☐ Satisfied ☐ Neither satisfied or dissatisfied ☐ Dissatisfied ☐ Very dissatisfied

3. Have you ever participated in a health research study?

☐ No ☐ Yes

4. Have you ever heard anything about 'electronic health records'?

By 'electronic health records' we mean computer records with complete and detailed health information, as described on the cover page of this survey.

- ☐ No PLEASE SKIP TO QUESTION 3
- ☐ Yes

If yes, where have you heard about electronic health records before? *Please X all that apply*

- ☐ The media (e.g. newspaper, radio, television)
- ☐ The internet (e.g. Wikipedia, Google)
- ☐ The NHS (GP, NHS letter, NHS website)
- ☐ Word of mouth (e.g. friend or relative)
- ☐ Another source ____________________

Next, we would like to know what you think about *electronic health records* .

5. *If your name and address were present*, should these groups have access to your 'Complete record', 'Partial record' or Neither record?

*By '**Complete record**' we mean all of your **detailed** health information (e.g. complete health history)*
*By '**Partial record**' we mean information will be **limited** to a specific purpose (e.g. prescriptions, allergies, etc.)*

	Complete Record	Partial Record	Neither Record
Doctors and nurses (e.g. deciding your treatment)	☐	☐	☐
Pharmacists (e.g. giving you medicines)	☐	☐	☐
GP receptionists (e.g. booking appointments)	☐	☐	☐
Ambulance and emergency department staff (e.g. responding to an emergency)	☐	☐	☐
Other health professionals (e.g. physiotherapists)	☐	☐	☐
You (e.g. reading your own record)	☐	☐	☐

6. If there was a national electronic health records system, would you want your record to be part of it for your *own healthcare*?

☐ Yes, complete record ☐ Yes, partial record ☐ No

7. Now thinking about your '*complete record*', should these groups have access to your record with your name and address present, removed or not at all ?

	Name & address Present	Name & address Removed	Not at all
NHS managers (e.g. plan health services)	☐	☐	☐
Health policy makers (e.g. check drug safety)	☐	☐	☐

8. If there was a national electronic health records system, would you want your record to be part of it for *health services planning and policy*?

☐ Yes, name & address present ☐ Yes, name & address removed ☐ No

9. Again thinking about your '*complete record*', should these groups have access to your record with your name and address present, removed or not at all ?

	Name & address Present	Name & address Removed	Not at all
NHS researchers (e.g. healthcare research)	☐	☐	☐
Academic health researchers (e.g. public health research)	☐	☐	☐
Health charities (e.g. cancer research)	☐	☐	☐
Drug companies (e.g. drug research)	☐	☐	☐

10. If there was a national electronic health records system, would you want your record to be part of it for *health research*?

☐ Yes, name & address present ☐ Yes, name & address removed ☐ No

11. If your record was part of a national electronic records system, would you worry about the security of your record?

☐ Yes ☐ No

12. Do you think the NHS is presently able to make electronic health records secure?

☐ Yes ☐ No

13. How do you feel about the security of electronic health records compared to your current health records ?

☐ Less secure ☐ Equally secure ☐ More secure

14. Overall, what do you think about the level of possible risks to the security of a national electronic health records system?

☐ Low risks ☐ Moderate risks ☐ High risks

15. If your record was part of a national electronic records system, would you expect to be asked before your records were accessed for any reason?

	Yes	No
... if your name and address were **<u>present</u>**?	☐	☐
... if your name and address were **<u>removed</u>**?	☐	☐

16. Overall, are you in favour of the development of a national electronic health records system?

☐ Yes ☐ No ☐ Undecided

People's opinions about electronic health records may depend on how much healthcare they use and why. *Remember we do not have your name or any other details.*

17. Do you have *any* of the following *long term health conditions*? *Please X all that apply*

- ☐ No health conditions
- ☐ High blood pressure
- ☐ Heart disease
- ☐ Diabetes
- ☐ Chronic bronchitis or emphysema
- ☐ Asthma
- ☐ Anxiety or depression
- ☐ Back problems and/or arthritis
- ☐ Fibromyalgia
- ☐ Migraine headaches
- ☐ Effects of stroke
- ☐ Cancer
- ☐ Alzheimer's disease or other dementia
- ☐ Bowel disorder
- ☐ Epilepsy
- ☐ Problems related to alcohol or drugs
- ☐ Thyroid condition
- ☐ Urinary incontinence
- ☐ Sexually transmitted disease
- ☐ HIV/AIDS
- ☐ Cataracts
- ☐ Glaucoma
- ☐ Other ________
- ☐ Prefer not to say

18. Including today, which healthcare services have *you* used in the past 6 months?

Please X all that apply

- ☐ No healthcare use
- ☐ Called an NHS telephone line (e.g. NHS Direct)
- ☐ General Practice (GP)
- ☐ GP out of hours service
- ☐ A walk-in or out of hours clinic
- ☐ Hospital - <u>emergency visit</u>
- ☐ Hospital - <u>planned overnight visit</u>
- ☐ Hospital - <u>planned day visit</u>
- ☐ Hospital - <u>outpatients visit</u>
- ☐ Other ________
- ☐ Prefer not to say
- ☐ Don't Know

19. Including today, how many times have *you* used *any* healthcare service in the past 6 months?

- ☐ No health care use
- ☐ 1-2 times
- ☐ 3-5 times
- ☐ 6-9 times
- ☐ 10 or more times
- ☐ Prefer not to say
- ☐ Don't know

People's opinions may also depend on whether they go with someone else to get healthcare. The next two pages are about people that you look after and the healthcare they use.

20. Do you have any dependent children?

- ☐ Yes
- ☐ No PLEASE SKIP TO QUESTION 23
- ☐ Prefer not to say PLEASE SKIP TO QUESTION 23

21. *If you answered yes to question 20*, to which healthcare services have *you accompanied* your dependent children in the past 6 months? *Please X all that apply*

- ☐ Did not need to accompany to any service
- ☐ Called an NHS telephone line (e.g. NHS Direct)
- ☐ General Practice (GP)
- ☐ GP out of hours service
- ☐ A walk-in or out of hours clinic
- ☐ Hospital - emergency visit
- ☐ Hospital - planned overnight visit
- ☐ Hospital - planned day visit
- ☐ Hospital - outpatients visit
- ☐ Other ____________________
- ☐ Prefer not to say
- ☐ Don't Know

22. *If you answered yes to question 20*, how many times have *you accompanied* your dependent children to *any* healthcare service in the past 6 months?

- ☐ Did not need to accompany to any service
- ☐ 1-2 times
- ☐ 3-5 times
- ☐ 6-9 times
- ☐ 10 or more times
- ☐ Prefer not to say
- ☐ Don't know

23. **Are you a carer for someone who is ill, frail, elderly or disabled? Please do not count anything that you are employed to do.**

- ☐ Yes
- ☐ No PLEASE SKIP TO QUESTION 26
- ☐ Prefer not to say PLEASE SKIP TO QUESTION 26

24. ***If you answered yes to question 23*, to which healthcare services have *you accompanied* the person(s) you care for in the past 6 months?** *Please X all that apply*

- ☐ Did not need to accompany to any service
- ☐ Called an NHS telephone line (e.g. NHS Direct)
- ☐ General Practice (GP)
- ☐ GP out of hours service
- ☐ A walk-in or out of hours clinic
- ☐ Hospital - emergency visit
- ☐ Hospital - planned overnight visit
- ☐ Hospital - planned day visit
- ☐ Hospital - outpatients visit
- ☐ Other ____________
- ☐ Prefer not to say
- ☐ Don't Know

25. ***If you answered yes to question 23*, how many times have *you accompanied* the person(s) you care for to *any* healthcare services in the past 6 months?**

- ☐ Did not need to accompany to any service
- ☐ 1-2 times
- ☐ 3-5 times
- ☐ 6-9 times
- ☐ 10 or more times
- ☐ Prefer not to say
- ☐ Don't know

Finally, we would like to know a bit about you to compare your answers to other people's responses.

26. Are you?

- ☐ Female
- ☐ Male
- ☐ Prefer not to say

27. What year were you born?

☐☐☐☐ e.g. 1965

28. What is your highest level of education?

- ☐ No academic qualification
- ☐ Left school at age 16 (e.g. GCSE)
- ☐ Left school at age 18 (e.g. A-Levels)
- ☐ Vocational Qualification (e.g. technical college)
- ☐ Degree
- ☐ Higher degree
- ☐ Prefer not to say

29. What is your ethnicity?

- ☐ Asian/Asian British
- ☐ Black/African/Caribbean/British Black
- ☐ Mixed/Multiple ethnic origins
- ☐ White British
- ☐ White Non-British
- ☐ Other ethnic group
- ☐ Prefer not to say

30. How confident are you using computers?

- ☐ Very confident
- ☐ Confident
- ☐ Fairly confident
- ☐ Not confident
- ☐ Prefer not to say

31. Where do you live?

- ☐ Kensington and Chelsea
- ☐ Wandsworth
- ☐ Hammersmith and Fulham
- ☐ Westminster
- ☐ Other Borough
- ☐ Prefer not to say

Thank you for your time

Please hand back your survey to the research team .
